# CD5 Controls Gut Immunity by Shaping the Cytokine Profile of Intestinal T Cells

**DOI:** 10.3389/fimmu.2022.906499

**Published:** 2022-06-02

**Authors:** Cornelia Schuster, Badr Kiaf, Teri Hatzihristidis, Anna Ruckdeschel, Janice Nieves-Bonilla, Yuki Ishikawa, Bin Zhao, Peilin Zheng, Paul E. Love, Stephan Kissler

**Affiliations:** ^1^ Joslin Diabetes Center, Harvard Medical School, Boston, MA, United States; ^2^ Section on Hematopoiesis and Lymphocyte Biology, Eunice Kennedy Shriver National Institute of Child Health and Human Development, National Institutes of Health, Bethesda, MD, United States; ^3^ Rudolf Virchow Center for Experimental Biomedicine, Wurzburg, Germany

**Keywords:** T cell, costimulation, autoimmunity, cytokines, mouse model

## Abstract

CD5 is constitutively expressed on all T cells and is a negative regulator of lymphocyte function. However, the full extent of CD5 function in immunity remains unclear. CD5 deficiency impacts thymic selection and extra-thymic regulatory T cell generation, yet CD5 knockout was reported to cause no immune pathology. Here we show that CD5 is a key modulator of gut immunity. We generated mice with inducible CD5 knockdown (KD) in the autoimmune-prone nonobese diabetic (NOD) background. CD5 deficiency caused T cell-dependent wasting disease driven by chronic gut immune dysregulation. CD5 inhibition also exacerbated acute experimental colitis. Mechanistically, loss of CD5 increased phospho-Stat3 levels, leading to elevated IL-17A secretion. Our data reveal a new facet of CD5 function in shaping the T cell cytokine profile.

## Introduction

CD5, one of the earliest markers used to identify T cells (1, 2), is a 67 kD transmembrane molecule expressed on the surface of all T cells and on a subset of B cells (3). Several CD5 ligands have been proposed, though none have yet been independently validated (4–9). CD5 may in fact have functionality independent of its extra-cellular domain (10). Early experiments ascribed to CD5 a costimulatory function because anti-CD5 could synergize with other stimuli to activate T cells *in vitro* (11–14). However, experiments using CD5 knockout (KO) mice demonstrated that CD5 is a negative regulator of thymocyte stimulation (10, 15–17). CD5 deficiency facilitates the positive selection of poorly selected thymocytes and increases negative selection of high-avidity clones (16, 17). This inhibitory role was extended to mature T cells, though exactly what signaling pathways CD5 co-opts to regulate T cell activation is still unclear (18). Along with its negative effect on T cell stimulation, CD5 was shown to diminish activation-induced cell death (AICD) (19, 20) through its interaction with casein kinase 2 (CK2) (21). CD5 levels are frequently used as a surrogate for TCR avidity, because CD5 expression is induced by TCR stimulation and CD5 surface levels are proportional to TCR signal intensity. Also, CD5 surface expression on resting T cells is proportional to a clone’s TCR affinity for its positively selecting self-peptide (17, 22). In addition, CD5 has been implicated in the extra-thymic differentiation of Foxp3^+^ regulatory T cells (Tregs) (23). In B cells, CD5 was reported to promote IL-10 expression (24, 25). Collectively, the functions ascribed to CD5 suggest an important role in immune regulation, yet germline CD5 KO causes no apparent immune pathology (15, 26). To further explore CD5 function, we generated inducible CD5 knockdown (KD) mice in the nonobese diabetic (NOD) mouse strain prone to autoimmunity (27). Loss of CD5 in NOD mice caused spontaneous wasting disease and severely exacerbated experimental colitis. Our data show that CD5 controls gut immunity by modulating the cytokine profile of intestinal T cells.

## Materials and Methods

### Mice

CD5 KD mice were generated by lentiviral transgenesis in the NOD mouse strain as described previously (28). Target sequences for CD5 KD by RNAi were ggatctccgtggtctatat (shRNA1) and ggagctgtgtctcactaca (shRNA2). All experiments shown were performed with the CD5 shRNA2 transgenic line. WT NOD mice used for comparison in all experiments were bred and housed in the same facility and in the same room as transgenic mice. NOD Rag KO, NOD TCRβ KO and NOD IgM KO were purchased from Jackson Laboratories, and bred with CD5 KD mice. C57BL/6 CD5 KD mice were generated by crossing CD5 KD NOD mice with C57BL/6 mice purchased from Jackson Laboratories. CNS1 KO NOD mice were generated by CRISPR/Cas9 genome editing as described earlier (29). NOD mice carrying a tamoxifen-inducible CD5 deletion by combination of floxed CD5 alleles with a Cre-ER^T2^ transgene were generated by backcrossing of C57BL/6 CD5 iKO mice described previously (30). Mice carrying a tamoxifen-inducible Cre-ER^T2^ recombinase transgene were obtained from Jackson laboratories. All experimental procedures in animals were approved by the Regional Government of Lower Franconia, Germany, or by Joslin’s Institutional Animal Care and Use Committee (protocol #2014-01), for experiments performed at the University of Wurzburg and at the Joslin Diabetes Center, respectively.

### CD5 Knockdown and Knockout

For gene knowdown in CD5 KD mice, doxycycline was added to the drinking water at 200 μg/ml, starting at 3 weeks of age (after weaning) unless otherwise indicated. Dox-treated CD5 KD mice are denoted as CD5 KD^dox^ throughout the manuscript, unless otherwise specificied. Mice had free access to doxycycline water, and bottles were changed twice per week. All WT mice used as controls for CD5 KD^dox^ were treated with doxycycline for the same duration and at the same dose. For gene knockout in the CD5^flox/flox^ x Cre-ER^T2^ model (inducible CD5 KO), mice were injected intraperitoneally with 1.5 mg tamoxifen dissolved in 150 μl corn oil every other day for 10 days, totalling 5 injections. CD5 knockdown and knockout were validated by cell surface staining for CD5 by flow cytometry (see below for details).

### Diabetes Frequency

Disease studies were performed with age-matched, contemporary cohorts of mice. Onset of diabetes was monitored by weekly measurements of glycosuria using Diastix (Bayer). Mice with two consecutive readings > 250 mg/dL were considered diabetic.

### Generation of Bone-Marrow Chimeras

For the generation of bone-marrow chimeras, NOD Rag KO mice were irradiated (800 rad) and injected intravenously with lineage-depleted bone-marrow cells (5x10^5/^mouse) from CD5 KD NOD mice. Alternatively, a mixture of lineage-depleted bone-marrow cells from NOD TCR KO and IgM KO mice (each either WT or CD5 KD) was injected into irradiated NOD Rag KO. All recipients were dox-treated. Lineage depletion was performed using magnetic separation with a MACS lineage-depletion kit (Miltenyi Biotech) according to the manufacturer’s instructions.

### Isolation of Lymphocytes

Blood was collected from the tail vein and lymphocytes were stained for flow cytometry after red blood cell lysis. Single cell suspensions were prepared from spleen and lymph nodes by mechanical disruption of tissue followed by red blood cell lysis using ACK buffer. Lamina propria cells were separated from intraepithelial lymphocytes using EDTA followed by digestion with Collagenase Type VIII (Sigma Aldrich) and DNAse (Roche). Lymphocytes were collected using a Percoll (GE Healthcare) density gradient. Peyer’s Patches were digested with Dispase II (Sigma-Aldrich) and DNAse (Roche).

### Isolation of Epithelial Cells

After removal of fat tissue, feces and Peyer’s patches, the intestine was extensively rinsed with HBSS (Ca^2+^/Mg^2+^ free) containing 10mM HEPES, then washed at 37 C (with shaking; 350 rpm) in HBSS (Ca^2+^/Mg^2+^ free) containing DTT 1mM, then washed at 37 C (with shaking; 350 rpm) in HBSS (Ca^2+^/Mg^2+^ free) containing 0.5mM EDTA. Harvested cells were stained with anti-CD45 and anti-EpCam antibodies. Epithelial cells identified as CD45^-^EpCam^+^ were sorted using a FACSAriaIII instrument (BD Biosciences) and further analyzed by qPCR.

### Naïve CD4T Cell Transfer

CD4^+^ T cells were purified from CD5 KD Thy1.2 and WT Th1.1 congenic NOD mice pre-treated with doxycycline. Cells were isolated and purified from the spleen and mesenteric lymph nodes using Naive CD4+ T Cell Isolation Kit (Miltenyi Biotec) according to the manufacturer’s instructions. After purification, 2 million cells in 100 μl of PBS were intravenously injected into NOD Rag^KO^ mice. 11 days later, mice were sacrificed, and CD4^+^ T cells Thy1.1 or Thy1.2 cells were analyzed by flow cytometry or qPCR.

### *In Vivo* Analysis of Intestinal Permeability

Gut leakiness was evaluated by the intestinal permeability of FITC-dextran 4 kD. Waterfasted mice (4-6h) were gavaged with FITC-dextran (600 mg/kg body weight, 120 mg/ml; Sigma-Aldrich). After 0.5 to 4 h, blood was collected and centrifuged at 4°C, 10,000 rpm, for 10 min. Plasma, half diluted in PBS, was analyzed for FITC-dextran 4 kD concentration with a fluorescence spectrophotometer (GloMax Discover plate reader, Promega) at excitation and emission wavelengths of 485 nm and 535 nm, respectively. Standard curves for calculating the FITC-dextran 4 kD concentration in the samples were obtained by diluting FITC-dextran 4 kD in PBS.

### Th17 and Th22 Cell Differentiations

CD4^+^ T cells were purified from CD5 KD or WT NOD mice (the mice were on Doxycycline for at least 2 months). Cells were isolated and purified from the spleen and mesenteric lymph nodes using Naive CD4+ T Cell Isolation Kit (Miltenyi Biotec) according to the manufacturer’s instructions. Naive CD4+ T cells were cultured in 6-well plates at a concentration of 10^6^ cell per ml of media. Cells were activated with 2 μg/mL plate-bound mouse anti-CD3 (clone 145-2C11; BioLegend) and 2 μg/mL soluble anti-CD28 (clone 37.51; BioLegend) in complete RPMI medium supplemented with 10% fetal bovine serum, 2 mM L-glutamine, 100 U/mL penicillin, 100 mg/mL streptomycin, 50 mM 2-b-mercaptoethanol, HEPES, pyruvate and NEAA. For Th17 cell cultures, recombinant mouse IL-6 (30 ng/mL), IL-1β (10 ng/mL), TGFβ (1 ng/mL), anti-IFN γ (5 ug/mL; clone XMG1.2; BioLegend) and anti-IL-4 (5ug/mL; clone 11B11; BioLegend) were added at the start of culture. 2-3 days later the cells were re-plated in a 24-well plate in complete RPMI with recombinant IL-6 (30 ng/mL), IL-1β (10 ng/mL), TGFβ (1 ng/mL), anti-IFN γ (5 ug/mL; clone XMG1.2; BioLegend) and anti-IL-4 (5ug/mL; clone 11B11; BioLegend) for additional 2-3 days. For Th22 cell cultures, recombinant mouse IL-6 (50 ng/mL), IL-23 (50 ng/mL), anti-IFN γ (5 ug/mL; clone XMG1.2; BioLegend) and anti-IL-4 (5ug/mL; clone 11B11; BioLegend) were added at initiation of culture. 2-3 days later the cells are re-cultured in a 24-well plate in complete RPMI with recombinant mouse IL-6 (50 ng/mL), IL-23 (50 ng/mL), anti-IFN γ (5 ug/mL; clone XMG1.2; BioLegend) and anti-IL-4 (5ug/mL; clone 11B11; BioLegend) for additional 2-3 days. All cytokines and blocking antibodies were from BioLegend. For IL-17A and IL-22 production analysis by flow cytometry or ELISA, recombinant mouse IL-23 (100 ng/mL) was added overnight to the cultures prior to measurement.

### Flow Cytometry

Flow cytometry measurements were performed using a LSRII or LSR Fortessa instrument (BD Biosciences). Cell sorting was performed with a FACSAriaIII (BD Biosciences) or MoFlo high-speed sorter. Data were analysed with FlowJo software (TreeStar Inc.). Fluorescently conjugated antibodies were purchased from Biolegend, eBioscience/Thermo Fisher Scientific and BD Biosciences. Intracellular staining was performed with a Cytofix/Cytoperm Plus Kit (BD Biosciences) or a FoxP3/Transcription Factor Staining Buffer Set (eBioscience/Thermo Fisher Scientific). Surface staining was performed in PBS and cells were pre-incubated with the CD16/32 Fc-receptor blocking antibody prior to labeling with fluorescently-conjugated antibodies. For exclusion of dead cells, a Zombie Fixable Viability Dye was used (Biolegend).

For *ex vivo* intracellular cytokine staining, cells were stimulated for 4h with PMA (Sigma-Aldrich, 50ng/ml) and ionomycine (1μg/ml) in the presence of Golgi-Stop (BD Biosciences) prior to flow cytometry staining.

### *In Vivo* T Cell Depletion Experiments

Mice were injected intraperitoneally with an α-CD4 or an α-CD8 antibody (200μg/mouse, Biolegend). Two consecutive injections were performed on day 1 and day 10. T cell depletion efficiency was measured in the blood by flow cytometry. Mice were monitored for weight loss by weekly weight measurements.

### Colitis Experiments

For T cell transfer colitis, CD4^+^ T cells were pre-sorted from spleens of NOD WT or CD5 KD mice using CD4 microbeads (Miltenyi Biotec). CD4^+^ CD45RB^hi^ T cells were then FACS-sorted and injected intravenously into NOD Rag KO mice. Recipient mice were treated with doxycycline and monitored for colitis symptoms weekly. Colitis scores in the transfer model were applied as follows (31):

1. Scattered inflammatory cell infiltrates in the mucosa
2. Diffuse mucosal infiltrates, no submucosal spreading with intact epithelium
3. Moderate inflammatory cell infiltrates into the mucosa and submucosa with epithelial hyperplasia and goblet cell loss
4. Marked inflammatory cell infiltration in mucosa and submucosa with crypt abcesses, goblet cell and crypt loss
5. Marked inflammatory cell infiltration in mucosa and submucosa with crypt loss and hemorrhage
Colitis severity was calculated using a combined score including the following criteria: Weight loss, colon length, macropathology (diarrhea, bloody stool) and histological scores.

DSS-induced colitis experiments were performed using 2.5% Dextran Sulfate Sodium (MP Biomedicals) in the drinking water. Mice were monitored for colitis symptoms daily. In the colon, histological scores in the DSS model were applied as follows (31):

1. Mild mucosal inflammatory cell infiltrates with intact epithelium
2. Inflammatory cell infiltrates into mucosa and submucosa with intact epithelium
3. Mucosal inflammatory cell infiltrates with focal ulceration
4. Inflammatory cell infiltrates into mucosa and submucosa and focal ulceration
5. Moderate cell infiltration into mucosa and submucosa with extensive ulceration
6. Transmural inflammation and extensive ulceration

Colitis severity was calculated using a combined score including the following criteria: Weight loss, colon length, macropathology (diarrhea, bloody stool) and histological scores.

In the small intestine, histological scores in the DSS model were calculated using the following pathological features: mucosal infiltrates, submucosal infiltration, villus blunting, swollen villus tips/edematous tips/hyperplasia and distorted villus structure/erosion. A combined small intestine score was calculated including histology scores, macropathology (diarrhea, bloody stool) and small intestine length.

### Cytokine/Chemokine Measurements

Serum cytokines/chemokines were analyzed using a multiplex assay kit (MCYTOMAG-70K MILLIPLEX Map Mouse Cytokine/Chemokine Magnetic Bead Panel) on a Milliplex ^®^ Analyzer (Millipore). ELISA was performed using the ELISA MAX™ Deluxe Set Mouse IL-17A, IL-22 or IL-10 from BioLegend (#432504, # 436304 and # 431414) following the manufacturer’s instructions.

### Histology

Tissue for histological analysis was fixed with buffered Formalin (Fisher Scientific) *in situ* followed by Fixation in Bouin’s Solution (Sigma-Aldrich). Paraffin embedded sections were prepared and stained with hematoxylin and eosin. Images were acquired using an Olympus BX-60 microscope equipped with an Olympus DP70 camera using the DPManager software. Pictures were processed using ImageJ software.

### List of Antibodies

**Table:** 

Antibody	Clone	Vendor
CD4-PerCP/Cy5.5	RM4-5	Biolegend
CD4-BV785	RM4-5	Biolegend
CD4-BV605	RM4-5	Biolegend
B220-BV605	RA3-6B2	Biolegend
CD19-ACP/Cy7	6D5	Biolegend
Nrp-1-PECy7	3E12	Biolegend
CD44-PECy7	IM7	Biolegend
CD44-APC/Cy7	IM7	Biolegend
CD62L-APC	MEL-14	Biolegend
CTLA-4-BV421	UC10-4B9	Biolegend
RORγt-Alexa Fluor 647	Q31-378	BD Biosciences
Foxp3-PE	MF-14	eBioscience/Thermo Fisher
IFNγ-APC	XMG1.2	Biolegend
Rat IgG1-APC Isotype control	RTK2071	Biolegend
Rat IgG2b, K-PE Isotype control	400608	Biolegend
Mouse IgG2a Alexa Fluor 647 Isotype control	G155-178	Biolegend
CD8a-APC	53-6.7	Biolegend
CD3-BV785	17A2	BioLegend
CD5-PE/Cy7 or BV510	53-7.3	BioLegend
IL-10-PE	JESS-16E3	ThermoFisher
IL-17A-PE or PE/Cy7	TC11-18H10.1	BioLegedn
IL-22	IL22JOP	ThermoFisher
IL-21-PE/Cy7	mhalx21	ThermoFisher
IL-21-PE/Cy5	4A9	BioLegend
IL-4-BV421	11B11	BioLegend
Thy1.1-AF700	OX-7	BioLegend
Thy1.2-PercP/Cy5.5	53-2.1	BioLegend
tSTAT3-PE	4G4B45	BioLegend
pSTAT3 (Tyr705)-PE	13A3-1	BioLegend
pSTAT3 (Tyr705)-APC	LUVNKLA	ThermoFisher
pSTAT6-PE/EF610	CHI2S4N	ThermoFisher
pSTAT1-PerCP/Cy5.5	A15158B	BioLegend
IL-17A-PE or PE/Cy7	TC11-18H10.1	BioLegend
IL-22-APC	IL22JOP	BioLegend
PD-1-BV605	29F.1A12	BioLegend
CD8a-APC/Cy7	53-6.7	BioLegend
IL-21R-PE/Cy5	4A9	BioLegend
IL-6R-PE/Cy7	W18166A	BioLegend
IL-23R-APC	12B2B64	BioLegend
EpCAM-APC/Cy7	G8.8	BioLegend
CD45-APC/Cy7	30-F11	BioLegend
LAG-3- PE/Dazzle™ 594	C9B7W	BioLegend
pSTAT4-PE	4LURPIE	ThermoFisher
CD8a-BV711 or APC/Cy7	53-6.7	BioLegend
CD4-PerCP/Cy5.5 or BV785 or BV605	RM4-5	BioLegend
B220-BV605	RA3-6B2	BioLegend
CD19-ACP/Cy7	6D5	BioLegend
Nrp-1-PECy7	3E12	BioLegend
CD44-PECy7 or APC/Cy7	IM7	BioLegend
CD62L-APC	MEL-14	BioLegend
CTLA-4-BV421	UC10-4B9	BioLegend
RORγt-Alexa Fluor 647	Q31-378	BD Biosciences
Foxp3-PE	MF-14	ThermoFisher
IFNγ-APC	XMG1.2	BioLegend
Rat IgG1-APC Isotype control	RTK2071	BioLegend
Rat IgG2b, K-PE Isotype control	400608	BioLegend
Mouse IgG2a, K Alexa Fluor 647 Isotype control	G155-178	BioLegend
RORγt-Alexa Fluor 647	Q31-378	BD Biosciences
Foxp3-PE	MF-14	ThermoFisher
IFNγ-APC	XMG1.2	BioLegend
Rat IgG1-APC Isotype control	RTK2071	BioLegend
Rat IgG2b, K-PE Isotype control	400608	BioLegend
Mouse IgG2a, K Alexa Fluor 647 Isotype control	G155-178	BioLegend

### Statistical Analyses

Data were analysed with the Prism software (Graphpad). Diabetes frequency comparisons were carried out using the Gehan-Breslow-Wilcoxon test. Other comparisons were performed using a two-tailed t-test, Mann-Whitney test or Wilcoxon matched pair test, as indicated in the figure legends. P < 0.05 was considered significant. P values indicated by asterisks were as follows: * P < 0.05, ** P < 0.01, *** P < 0.001, unless otherwise indicated. Variances for t-test comparisons were determined with the Prism software, and were found not to be significantly different. Data met the assumption of the test used, i.e. that data were normally distributed and had similar variance. Sample sizes were approximated in initial experiments, and adjusted to increase power as needed in replicate experiments, based on initially observed effect size.

## Results

### Loss of CD5 in Autoimmune-Prone NOD Mice Causes Wasting Disease

**Figure 1:**
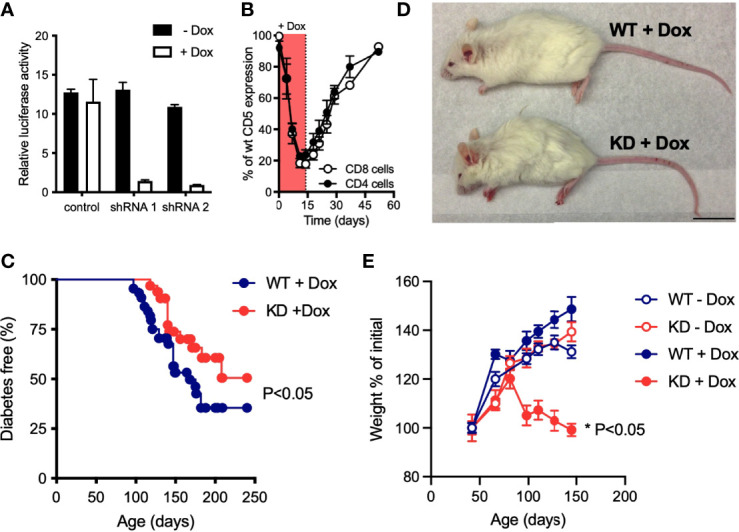
Loss of CD5 in NOD mice causes wasting disease **(A)** Doxycycline (dox)-inducible CD5 KD shRNA constructs were validated by luciferase reporter assay, using a dual-luciferase vector that incorporates the *CD5* cDNA (n=2-3 biological replicates). Data are representative of 4 experiments. **(B)** Relative CD5 expression in blood CD4^+^ or CD8^+^ T cells from CD5 KD mice (n=3) compared to WT NOD mice after dox-feeding followed by dox-removal from the drinking water. **(C)** Spontaneous diabetes frequency in cohorts of dox-treated CD5 KD (KD, n = 32) and WT (n = 44) NOD mice, P-values were calculated using the Gehan-Breslow-Wilcoxon test and statistical significance (P < 0.05) is indicated. **(D)** Representative picture of 5 months-old dox-treated CD5 KD and WT NOD mice. **(E)** Weight curves of CD5 KD and WT NOD mice with or without dox-treatment (n=17-21). *P < 0.05, two-tailed unpaired t-test.

Germline CD5 KO in the C57BL/6 (B6) background causes no obvious pathology (15), suggesting that CD5 may be dispensable for immune homeostasis. CD5 modulates the reactivity of both thymocytes (16) and of mature peripheral T cells (19). We hypothesized that the lack of a disease phenotype in CD5 KO mice could be due to compensation during thymic selection whereby CD5 deficiency caused the selection of T cell receptor (TCR) repertoire with lower affinity/avidity for positively selecting self-peptides. We reasoned that inducible CD5 repression would allow us to bypass any compensatory effects of thymic selection by inhibiting CD5 in post-selection T cells. We further speculated that a genetic background prone to autoimmunity such as the NOD mouse could accentuate the consequences of CD5 deficiency. We used lentiviral RNAi to generate mice with doxycycline-inducible CD5 knockdown (KD) (**Figure 1A**) in the NOD strain that develops spontaneous autoimmune diabetes. Doxycycline (dox) feeding caused CD5 levels to drop by up to 80% within a week. Upon dox removal, CD5 levels returned to near wild-type (WT) levels (**Figure 1B**). Continuous CD5 deficiency had a mild but statistically significant protective effect against autoimmune diabetes (**Figure 1C**). This is consistent with the protective effect of CD5 KO previously reported in experimental autoimmune encephalomyelitis (EAE) and associated with increased AICD (19). Unexpectedly, all dox-treated CD5 KD NOD mice (CD5 KD^dox^) developed wasting disease between 3 and 4 months of age (**Figures 1D, E**). Neither untreated CD5 KD or WT mice nor dox-treated WT (WT^dox^) mice showed any signs of weight loss or disease beyond the expected diabetes phenotype (**Figure 1E**). To test if the transgene insertion site or the difference between partial CD5 KD and complete CD5 KO could explain pathogenesis in CD5 KD NOD mice, we bred the lentiviral CD5 KD transgene from NOD mice into the B6 background. Strikingly, none of the CD5 KD NOD x B6 F1 offspring developed disease when treated with doxycycline (**Supplementary Figure 1**). Further backcrossing showed that CD5 KD was not pathogenic in B6 mice (**Supplementary Figure 1**). These data suggest that NOD mice are inherently prone to pathology following CD5 repression, likely due to their genetic susceptibility to immune dysregulation.

### CD5 Deficiency Leads to GALT Hyperplasia and Histological Anomalies in the Gut

**Figure 2:**
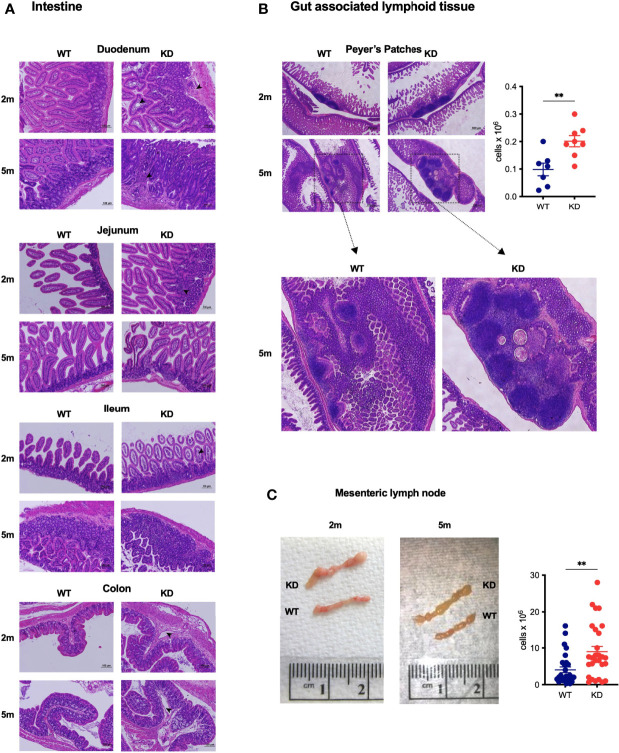
CD5 KD causes GALT hyperplasia and histological anomalies in the gut **(A, B)** Representative images of H&E stained histological sections of the intestine (duodenum, jejunum, ileum and colon) **(A)** or Peyer’s Patches **(B)** of dox-treated CD5 KD and WT NOD mice at 2 months (2m) or 5m of age. Scale bars are 100μm **(A)** or 500μm (b, upper panel). In b, the total cell number in Peyer’s Patches of 2m old dox-treated CD5 KD and WT NOD mice (n=7-8) is shown. *P < 0.05, two-tailed unpaired t-test. **(C)** Representative images of mesenteric lymph nodes (mLN) of dox-treated CD5 KD and WT NOD mice at 2m or 5m of age. The right panel shows total cell numbers in mLN of 2m old animals (n=28), **P < 0.01, two-tailed unpaired t-test.

We observed the expected insulitis characteristic for the NOD model of autoimmune diabetes (not shown) in CD5 KD^dox^ mice. The only other organ with obvious histological changes was the gut, including gut associated lymphoid tissues (GALT). CD5 KD^dox^ NOD mice had mild cell infiltrations, edematous structures and distorted or swollen villi in the small intestine. In the colon, CD5 KD^dox^ NOD mice displayed edematous structures (**Figure 2A**). Peyer’s patches (PP) and the mesenteric lymph nodes (mLN) were enlarged both prior to and after measurable weight loss (at 2 and 5 months, respectively, **Figures 2B, C**). Tissue hyperplasia was associated with increased cellularity (**Figures 2B, C**), though the overall distribution of lymphocyte populations in the PP and mLN was not substantially altered (**Supplementary Figure 2**). In contrast, we did not observe increased cellularity in the spleen of CD5 KD^dox^ mice at any time before or after disease onset (**Supplementary Figure 2**). These data suggested that wasting disease in CD5 KD^dox^ NOD mice was caused by changes in the gut immune compartment.

### Induced CD5 Deficiency Compromises Gut Barrier Integrity

**Figure 3:**
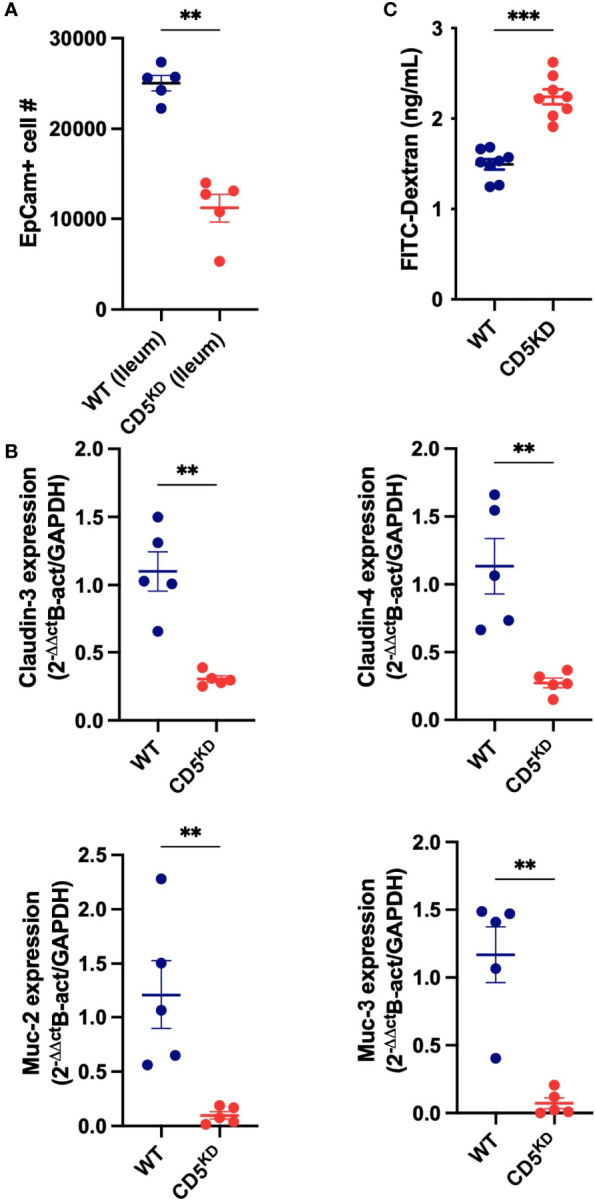
CD5 deficiency compromises gut barrier integrity **(A–C)** WT (black) or CD5 KD (red) NOD mice were treated with doxycycline for 2 months. **(A)** Number of epithelial cells (EpCam^+^) in the ileum of CD5 KD^dox^ and their littermate controls (n = 8 per group). This data represent cells analyzed in 60 seconds at the same flow rate by flow cytometry. **(B)** Relative mRNA levels for Muc2, Muc4, Cldn4 and Cldn4 in ileum epithelial cells of CD5 KD^dox^ and their littermate controls (n = 5 per group). Each symbol represents an individual mouse, lines indicate the mean). All statistical analyses were performed by two-tailed Mann–Whitney test. *P < 0.05, **P < 0.01, ***P < 0.001.

Given that CD5 KD^dox^ mice presented with abnormal gut histology and altered intestinal immune homeostasis, we evaluated the integrity of the gut epithelial barrier. We observed a significant decrease in the number of epithelial cells recovered from the colon of CD5 KD^dox^ mice (**Figure 3A**). This decrease was accompanied with reduced expression of several anti-bacterial peptides and mucus components in gut epithelial cells (**Figure 3B**). Significantly, the loss of CD5 caused higher serum concentration of dextran-FITC following oral gavage (**Figure 3C**), indicating loss of gut barrier integrity.

### Disease in CD5 KD NOD Mice Is Driven by Modified T Cell Function

**Figure 4:**
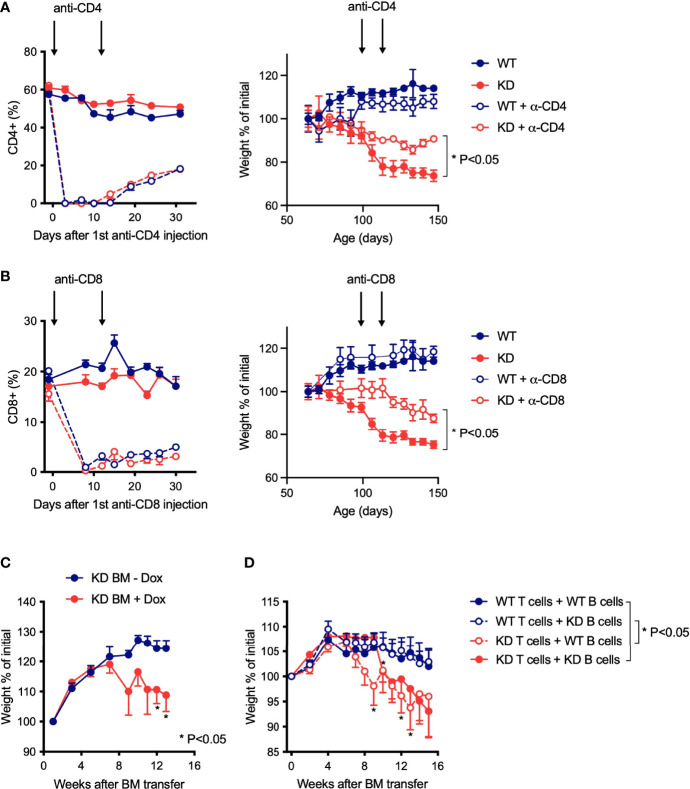
Disease in CD5 KD NOD mice is driven by modified T cell function **(A, B)** Antibody-mediated T cell depletion in dox-treated CD5 KD or NOD WT mice. Relative percentages of CD4^+^ **(A)** or CD8^+^ **(B)** T cells in the blood (left panels) and weight curves (right panels) of dox-treated CD5 KD and WT NOD mice with or without anti-CD4 antibody **(A)** or anti-CD8 antibody **(B)** treatment (arrows) (n=5-12). *P < 0.05 (two-tailed unpaired t-test). **(C, D)** Weight curves for bone marrow (BM) chimeric mice, shown as percent of initial weight. In c, NOD Rag KO mice (n=5-7) were reconstituted with CD5 KD BM and either treated or not with doxycycline. In D, NOD Rag KO mice (n=11-12) were reconstituted with a mixture of BM from WT or CD5 KD T-cell deficient (TCR KO) and B-cell deficient (IgM KO) mice, and all BM transplant recipients received dox-treatment. *P < 0.05 (two-tailed unpaired t-test).

We next asked if disease in CD5 KD^dox^ NOD mice was dependent on altered T cell function. Depletion of either CD4^+^ or CD8^+^ T cells after onset of weight loss was sufficient to ameliorate disease (**Figures 4A, B**). Although these results supported a role for T cells in pathogenesis, a subset of B cells also expresses CD5 and could be involved (3, 25). To explore whether both T and B cell dysfunction contributed to pathology, we performed mixed bone-marrow (BM) transplant experiments. We first established that BM from CD5 KD mice could transfer disease to irradiated NOD Rag knockout (KO) recipient mice treated with doxycycline (**Figure 4C**). We then combined BM from WT and CD5 KD T cell- and B cell-deficient mice (TCR KO and IgM KO, respectively) in all four combinations to confine CD5 KD to either T or B cells. This approach demonstrated that CD5 KD in T cells was necessary to cause disease (**Figure 4D**) unlike CD5 KD in B cells that had no deleterious effect. We concluded that CD5 KD in T cells was driving pathogenesis.

### The Frequency of Peripherally-Induced Tregs Is Decreased by CD5 KD, but Loss of pTregs Alone Is Not Pathogenic in NOD Mice

**Figure 5:**
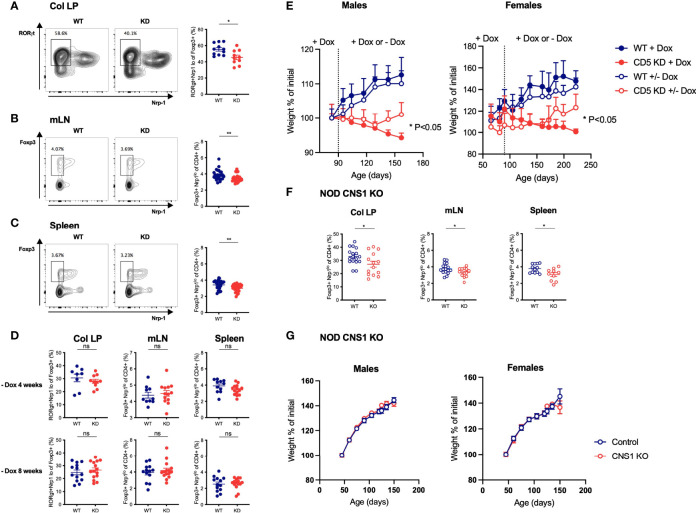
The frequency of peripherally-induced Tregs is decreased by CD5 KD, but loss of pTregs alone does not cause wasting disease **(A–C)** Lymphocytes from the colonic lamina propria (LP, a), mesenteric lymph nodes (mLN, b) and spleen **(C)** of dox-treated CD5 KD and WT mice were analyzed by flow cytometry. pTregs were characterized as RORγt^+^Nrp-1^lo^ cells gated within total CD4^+^Foxp3^+^ cells in the colonic lamina propria **(A)** and as Foxp3^+^Nrp-1^lo^ cells within total CD4^+^ cells in mLN and spleen **(B**, **C)**. All samples were gated on live CD4^+^CD8^-^ lymphocytes. Representative FACS plots are shown on the left, cell frequency data (mean ± SEM) on the right. All mice were 7 weeks old, n=26-27 for spleen, n=26 for mLN and n=10-11 for colonic LP. *P < 0.05 (two-tailed unpaired t-test). **(D)** CD5 KD and WT NOD mice were treated with dox for 4 weeks then kept without dox-treatment for another 4 or 8 weeks, as indicated. pTreg frequencies in the colonic lamina propria, mesenteric lymph nodes (mLN) and spleen were analyzed by flow cytometry. P-values were calculated by two-tailed unpaired t-test. ns: P > 0.05. **(E)** CD5 KD and WT NOD mice were treated with dox either throughout the experiment or fed with normal water (without dox) from 3m (left panel; n=5-10 male mice) or 2m of age (right panel; n=15-28 female mice). Weight curves are shown (mean ± SEM), *P < 0.05, **P < 0.01 (two-tailed unpaired t-test). **(F)** pTreg frequencies in the colonic LP, mLN and spleen of CNS1 KO and WT NOD mice, analyzed by flow cytometry as described above. *P < 0.05 (two-tailed unpaired t-test). **(G)** Weight curves (mean ± SEM) for CNS1 KO or WT NOD male (left panel, n=19-26) and female (right panel, n=16-29) mice. ns - not significant.

CD5 KO was reported to impair the extra-thymic differentiation of Foxp3^+^ Tregs (23). CD5 KO mice harbor fewer peripherally-induced Tregs (pTregs), characterized by low Neuropilin-1 (Nrp-1) expression, particularly in the gut where pTregs are most abundant (32). Because pTreg deficiency can cause gut inflammation (32–35), we speculated that CD5 KD may promote gut immune dysfunction by inhibiting pTreg formation. Consistent with published data, CD5 KD diminished pTreg frequency in the colonic lamina propria, the mLN and the spleen (**Figures 5A–C**). Notably, reinstating CD5 expression by stopping dox treatment caused the frequency of pTregs to return to WT levels within 4-8 weeks (**Figure 5D**). At the same time, cessation of dox-treatment resulted in significant weight gain in CD5 KD^dox^ mice (**Figure 5E**). The correlation of pTreg recovery with disease amelioration suggested that the loss of pTregs may be causal for disease in CD5 KD mice. We tested this hypothesis by monitoring the weight of CNS1 KO NOD mice (29) that have a pTreg deficiency similar to that observed in CD5 KD^dox^ NOD animals (**Figure 5F**). However, CNS1 KO NOD mice suffered neither the gut pathology nor the weight loss observed in CD5 KD^dox^ mice (**Figure 5G**). The data suggested that a decrease in pTregs alone was not pathogenic in CD5 KD^dox^ NOD mice.

### CD5 KD Exacerbates Experimental Colitis by Modifying the Function of Effector T Cells

**Figure 6:**
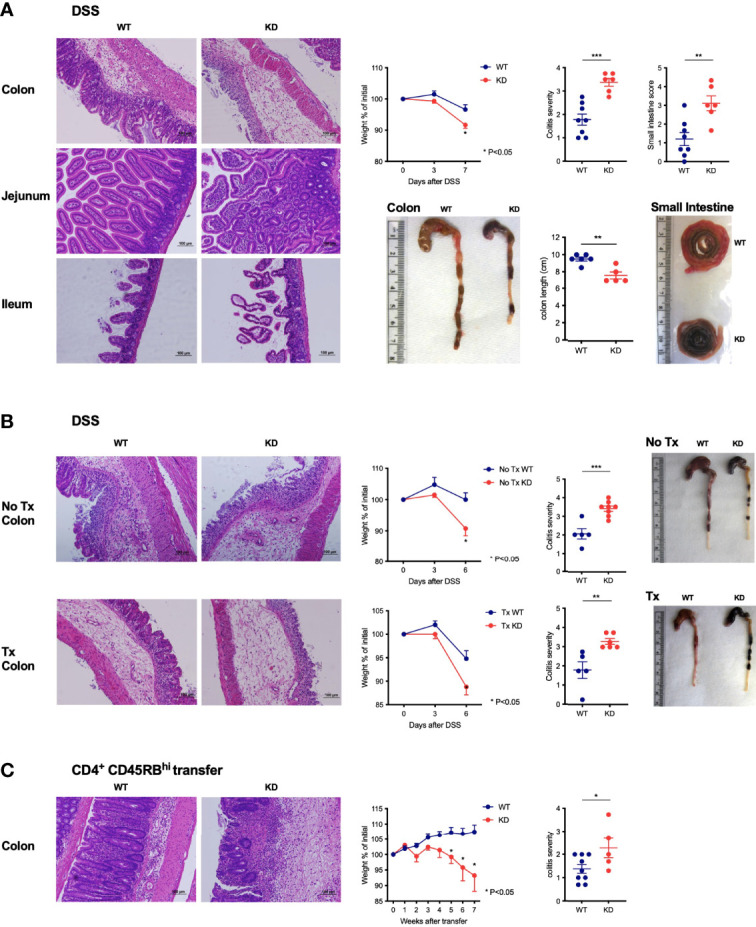
CD5 KD exacerbates colitis by modifying the function of effector T cells **(A)** DSS-induced colitis in dox-treated CD5 KD and WT NOD mice. Representative H&E stained colon and small intestine histology, weight curves depicting percent of initial weight, pathology scores and representative organ pictures are shown; n=6-8 mice per group. * P < 0.05 (two-tailed unpaired t-test). **(B)** DSS-induced colitis in thymectomized (lower panels) dox-treated CD5 KD and WT NOD mice. Controls include non-thymectomized mice (upper panels). Representative H&E stained colon histology, weight curves depicting percent of initial weight, pathology scores and representative organ pictures are shown; n=5-8 mice per group. **(C)** CD4^+^CD45RB^hi^ T cells were FACS-sorted from spleens of CD5 KD and WT NOD mice and injected intravenously into NOD Rag KO mice which were then treated with dox. Representative H&E stained colon histology, weight curves depicting percent of initial weight and colitis scores are shown; n=5-9 mice per group. *P < 0.05 (two-tailed unpaired t-test). **P < 0.01; ***P < 0.001 (two-tailed unpaired t-test). All scale bars are 100μm.

Our discovery that CD5 KD caused T cell-dependent wasting disease associated with gut immune dysregulation prompted us to ask if the loss of CD5 would exacerbate colitis in experimental models for inflammatory bowel disease (IBD). CD5 KD increased the severity of colitis in the dextran sodium sulfate (DSS)-induced colitis model (36) (**Figure 6A**). Strikingly, CD5 repression caused severe hemorrhagic inflammation in the small intestine in DSS fed mice (**Figure 6A**), a pathology that is not usually observed in DSS-treated WT mice. Germline CD5 deletion is known to modify T cell selection in the thymus (10, 16, 37). To evaluate if the increased severity of experimental colitis in CD5 KD^dox^ mice derived from altered thymic selection, we repeated DSS-colitis experiments in mice thymectomized prior to dox treatment. CD5 repression increased colitis severity in mice both with and without a thymus (**Figure 6B**), suggesting that CD5 silencing directly affected mature peripheral T cells. To further test this notion, we used the T cell-transfer model for IBD where disease is induced in immuno-compromised mice by transplantation of CD45RB^hi^ CD4^+^ T cells (38). Again, CD5 inhibition exacerbated colitis in this adoptive transfer model that is comparatively mild in the NOD strain (**Figure 6C**). We concluded that CD5 KD modifies intestinal T cell activity thereby severely exacerbating induced colitis.

### Loss of CD5 Increases the Propensity of T Cells to Secrete IL-17A

**Figure 7:**
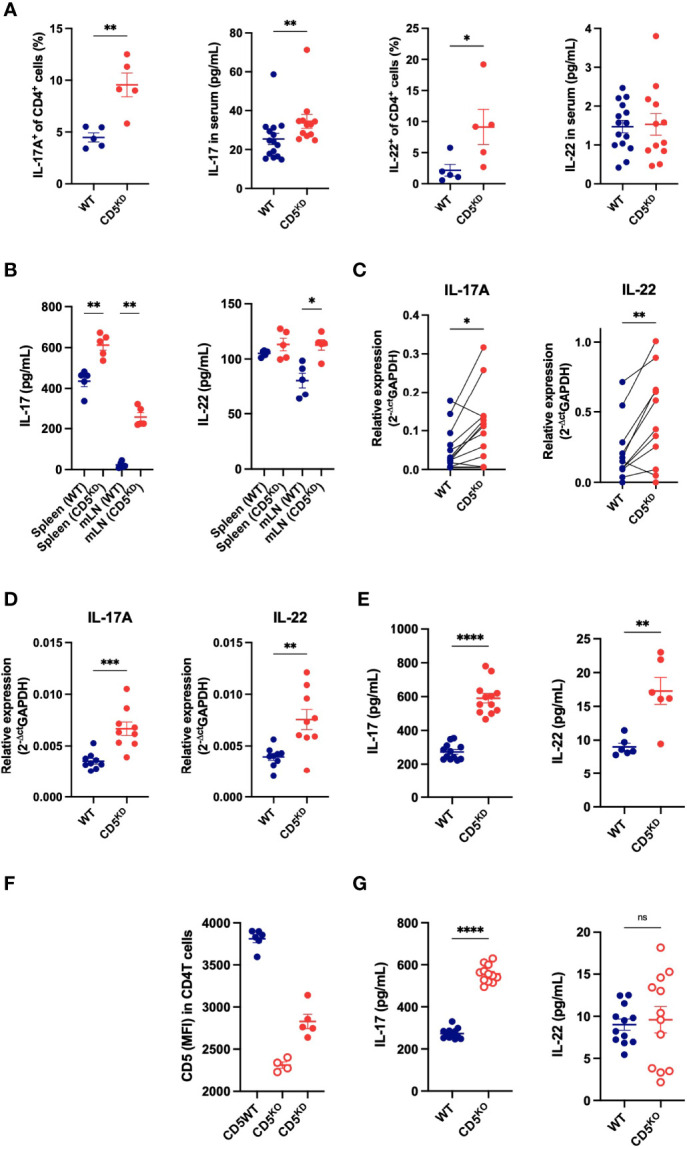
Loss of CD5 increases the propensity of T cells to secrete IL-17A. **(A)** Frequency of IL-17A^+^ and IL-22^+^ CD4^+^ T cell and serum concentrations of IL-17A and IL-22 in CD5 KD^dox^ (red) and WT (black) mice treated with doxycycline for 2 months (n = 5 mice per group). **(B)** IL-17A and IL-22 concentration in the culture medium of cells (mLN or spleen) after overnight IL-23 stimulation (100ng/mL) (n = 5 mice per group). **(C)** IL-17A and IL-22 mRNA levels in WT and CD5KD CD4^+^ T cells after adoptive transfer into gender-matched NOD.*scid* mice treated with doxycycline (paired samples from 11 mice). **(D, E)** IL-17A and IL-22 mRNA levels **(D)** and cytokine concentration in the culture medium from CD4^+^ T cells from WT^dox^ and CD5 KD^dox^ mice after *in vitro* Th17 and Th22 differentiation. (n = 6-12 technical replicates per group, starting material was pooled from 2-3 mice). **(F)** CD5 surface expression (MFI: mean fluorescence intensity) on CD4^+^ T cells from CD5 KD^dox^ (red), CD5KO (blue) and WT littermates analyzed by flow cytometry after 2 months of doxycycline (CD5 KD) or tamoxifen (CD5 KO) treatment. (n = 4-6 mice per group). **(G)** IL-17A and IL-22 concentration in the culture medium of WT and CD5KO CD4^+^ T cells after *in vitro* Th17 and Th22 differentiation. (n = 12 technical replicates per group, starting material was pooled from 2-3 mice). Data were compared by two-tailed Mann–Whitney test except in **(C)** where data were compared by two-tailed Wilcoxon matched-pairs test. *P < 0.05, **P < 0.01, ***P < 0.001, ****P < 0.0001, ns - not significant.

We next sought to understand how the effector T cell compartment was altered by CD5 repression. We found that CD5 KD increased the basal level of IL-17A^+^CD4^+^ T cells and of serum IL-17A levels (**Figure 7A**). A similar but less pronounced trend was observed for IL-22 producing T cells. Upon *in vitro* stimulation, CD5 KD^dox^ T cells from both spleen and mesenteric lymph nodes secreted significantly higher amounts of IL-17A and to a lesser extent IL-22 (**Figure 7B**). To confirm that CD5 deficiency caused an T cell intrinsic increase in IL-17A, and possibly IL-22, secretion by CD4^+^ T cells, we adoptively transferred congenically marked naive CD4^+^ T cells from WT and CD5 KD mice into immuno-deficient recipients. Transferred CD5 KD T cells again expressed significantly higher levels of IL-17A and IL-22 in comparison to WT cells in the same recipients after dox treatment (**Figure 7C**). Similarly, naive CD4 T cells stimulated under Th17 or Th22 skewing conditions produced elevated amounts of IL-17A and IL-22 at the transcriptional and protein level when CD5 expression was repressed (**Figures 7D, E**).

To further demonstrate that the loss of CD5 modifies the cytokine profile of CD4^+^ T cells, we used an alternative model for CD5 deletion where a floxed CD5 allele is combined with a Cre-ER^T2^ transgene in NOD mice, allowing CD5 deletion upon tamoxifen injection (30). This inducible CD5 knockout (CD5 KO) model caused a complete loss of CD5 expression (**Figure 7F**) and again increased IL-17A secretion in stimulated T cells (**Figure 7G**). Collectively, the data show that CD5 repression increases IL-17A expression, and to a lesser extent IL-22, in CD4^+^ T cells. Of note, CD5 deficiency did not significantly change IL-10 levels *in vitro* or *in vivo* or T cell-derived TNF-α *in vitro* (**Supplementary Figure 3**) that are also known to impact gut inflammation.

### CD5 Deficiency Increases Stat3 Activation

**Figure 8:**
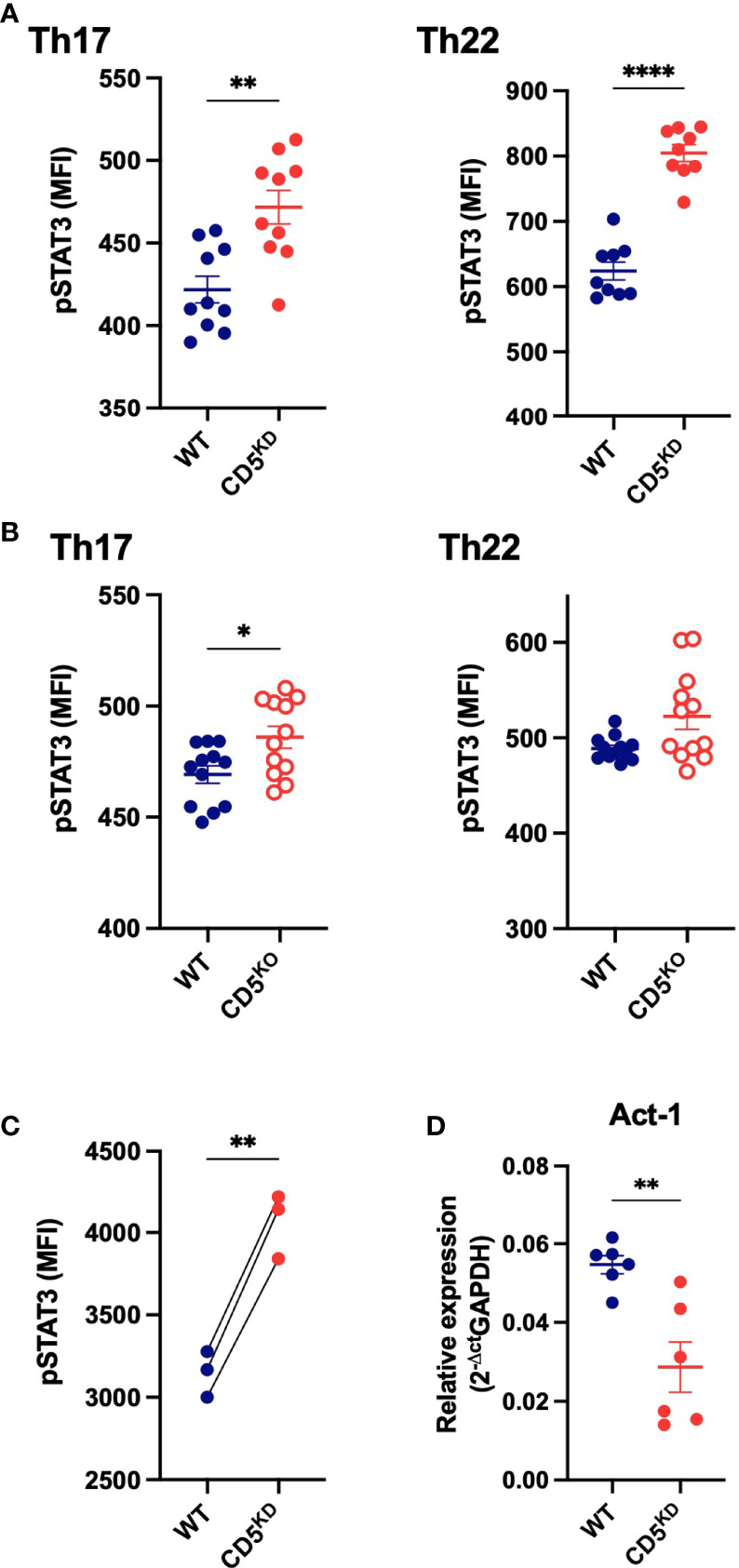
CD5 deficiency increases Stat3 phosphorylation. **(A–C)** Stat3 phosphorylation (Tyr705) levels in CD5 KD^dox^, CD5 KO and WT CD4^+^ T cells after *in vitro* Th17 and Th22 differentiation **(A, B)** and in WT and CD5 KD CD4^+^ T cells after adoptive transfer into gender-matched NOD.*scid* mice treated with doxycycline **(C)**. **(D)** Act-1 mRNA levels in WT^dox^ and CD5 KD^dox^ CD4^+^ T cells after *in vitro* Th17 differentiation. (n = 6 replicates per group). Data were compared by two-tailed Mann–Whitney test except in **(C)** where data were compared by two-tailed Wilcoxon matched-pairs test. *P < 0.05, **P < 0.01, ****P < 0.0001.

IL-17A is transcriptionally regulated by Stat3 that is activated by phosphorylation downstream of the receptors for IL-6 and IL-23. Neither of these cytokine receptors were up-regulated in CD5 KD^dox^ cells (not shown). However, we found that the loss of CD5 increased the levels of phospho-Stat3 after stimulation in both CD5 KD^dox^ and CD5 KO T cells (**Figures 8A, B**). Stat3 phosphorylation was also elevated basally in CD5 KD^dox^ T cells following adoptive transfer in comparison to WT^dox^ cells in the same recipient animals (**Figure 8C**). Exactly how CD5 deficiency leads to increased Stat3 phosphorylation is unclear, but we found that Act-1, recently described as an inhibitor of Stat3 activation (39), was decreased by CD5 KD (**Figure 8D**). The results suggest that CD5 deficient T cells are poised to express IL-17A owing to basally elevated phosphorylation of its key transcription factor Stat3.

## Discussion

CD5 was cloned over 30 years ago as one of the earliest T cell markers (3), yet the full extent of its function remains elusive (18, 26). A number of research groups have sought to understand CD5 function and identified several aspects of T cell biology impacted by CD5. Most notably, CD5 modifies thymocyte selection (16, 17), AICD (19) and extra-thymic Treg induction (23). Despite these functions, CD5 appeared to be largely dispensable to immune homeostasis, because germline CD5 KO mice do not develop any immune pathology (15). The data presented herein show that CD5 expression impacts IL-17A expression and thereby affects gut immunity. This role became apparent when CD5 was repressed in an autoimmune-prone mouse strain.

Basal CD5 expression is set during thymic positive selection, whereby relatively high TCR avidity for self-ligands leads to high constitutive CD5 levels and low TCR avidity to low CD5 levels (17, 40). But CD5 expression is dynamic throughout a T cell’s lifetime (41). In a striking example of CD5 adaptability, one model of antigen-specific tolerance strictly depended on CD5 up-regulation in mature peripheral T cells (42). The breakdown of gut immune tolerance observed in CD5 KD^dox^ NOD mice may thus derive in part from the inability of highly reactive T cell clones to modify their activity using CD5. Although the details of how CD5 deficiency causes gut T cells to become more inflammatory remain to be fully elucidated, it appears that the loss of CD5 causes an increase in steady-state Stat3 phosphorylation with an ensuing rise in basal IL-17A secretion in the CD4^+^ T cell compartment. A recent report by Singh and colleagues (30) described that CD5 modulates NF-κB activity by increasing IκK levels. NF-κB, together with Stat3, promotes IL-17A expression (43). The increase in IL-17A we observed in CD5 deficient T cells may therefore derive from both elevated NF-κB and Stat3 activity.

CD5 deletion had also been reported to diminish the peripherally-induced Treg compartment. This effect was replicated in CD5 KD^dox^ mice, as we did observe fewer pTregs in this model. We had previously generated CNS1 KO NOD mice that have comparable low levels of pTregs, but these mice did not suffer from similar wasting disease. We concluded that the loss of pTregs alone could not explain the pathology of NOD mice with CD5 KD. However, gut immune homeostasis is known to depend on the balance between inflammatory and regulatory cells, and of Th17 and Treg cells in particular (44). Consequently, it is plausible that the imbalance between a diminished pTreg compartment and an enlarged IL-17A-producing effector T cell population underlies the gut inflammation observed in CD5 KD^dox^ mice. In this context, Stat3 whose function is increased by CD5 deficiency had previously been described as a critical regulator of the Th17/Treg balance, particularly in inflammatory bowel disease (45). Moreover, a T cell-specific deletion of Stat3 had been shown to diminish the severity of DSS-colitis (46), consistent with our observation that increased Stat3 activity in CD5 KD T cells in turn exacerbates gut inflammation in this model. IL-17A was shown to be a key cytokine in colitis (47), further supporting our hypothesis that the increase in IL-17A secretion by CD5-deficient T cells, secondary to increased Stat3 phosphorylation, is causal for the disease phenotypes we observed in CD5 KD NOD mice.

While CD5 deficiency did not to impact gut immunity in C57BL/6 mice, it did have a severe effect in the NOD strain that is pre-disposed to autoimmunity. In this regard, it is of interest that CD5 has been identified as a candidate gene for a risk variant associated with Crohn’s disease, a disease that shares genetic risk with type 1 diabetes (48), the main trait of the NOD mouse model. We speculate that the loss of CD5 in combination with risk variants for autoimmunity in the NOD genome cause a Crohn’s-like pathology. Indeed, the regions of the gut affected in CD5 KD^dox^ mice differ from conventional colitis models. We observed pathology in all regions of the intestine, reminiscent of Crohn’s disease rather than ulcerative colitis which is confined to the colon. Most notably, DSS treatment caused severe enteritis only in the absence of CD5. These observations support a role for CD5 in Crohn’s disease as suggested by GWAS data (48).

Finally, it must be noted that the exact mechanism by which CD5 modifies T cell function remains to be fully understood. Many studies have examined intracellular signals downstream of CD5, yet there is no concensus as to which pathway(s) mediates this receptor’s effects. Even the most sophisticated and comprehensive methods used recently failed to unravel CD5’s exact function (18). Notwithstanding, CD5 would appear to play an extensive role in controlling T cell behavior that merits further investigation.

## Data Availability Statement

The raw data supporting the conclusions of this article will be made available by the authors, without undue reservation.

## Ethics Statement

The animal study was reviewed and approved by Joslin Diabetes Center IACUC.

## Author Contributions

CS designed and performed experiments, analysed and interpreted data and wrote the manuscript. BK designed and performed experiments, analyzed and interpreted data and edited the manuscript. TH designed and performed experiments, analyzed and interpreted data and edited the manuscript. JN-B and AR performed experiments and analysed data. YI, BZ and PZ performed experiments. PL interpreted data and edited the manuscript. SK conceived and supervised the project, designed experiments, interpreted data and wrote the manuscript. All authors contributed to the article and approved the submitted version.

## Funding

Supported by NIH grants P30DK036836 and S10OD021740), and the Dana-Farber/Harvard Cancer Center Rodent Histopathology Core for histological tissue preparation (supported by NCI Cancer Center Support grant P30CA06516). This project was funded in part by an NIAID grant (R21AI122110) to SK.

## Conflict of Interest

The authors declare that the research was conducted in the absence of any commercial or financial relationships that could be construed as a potential conflict of interest.

## Publisher’s Note

All claims expressed in this article are solely those of the authors and do not necessarily represent those of their affiliated organizations, or those of the publisher, the editors and the reviewers. Any product that may be evaluated in this article, or claim that may be made by its manufacturer, is not guaranteed or endorsed by the publisher.
