# Supplementary material for: CD5 Controls Gut Immunity by Shaping the Cytokine Profile of Intestinal T Cells
Source: Front Immunol. 2022 Jun 2;13:906499. doi: 10.3389/fimmu.2022.906499 (PMC9201032; doi:10.3389/fimmu.2022.906499)

Supplementary Figure 1: Loss of CD5 in NOD but not C57BL/6 mice causes wasting disease

(a) Weight curves for CD5 KD (KD, n=7) and WT (n=15) NOD mice treated with dox from birth. (b) Weight curves for CD5 KD and WT NOD x B6 F1 (left, n=5 for KD and n=4 for WT) and F7 B6-backcross (right, n=22 for KD and n=14 for WT). All mice were treated with dox starting at 3 weeks of age.

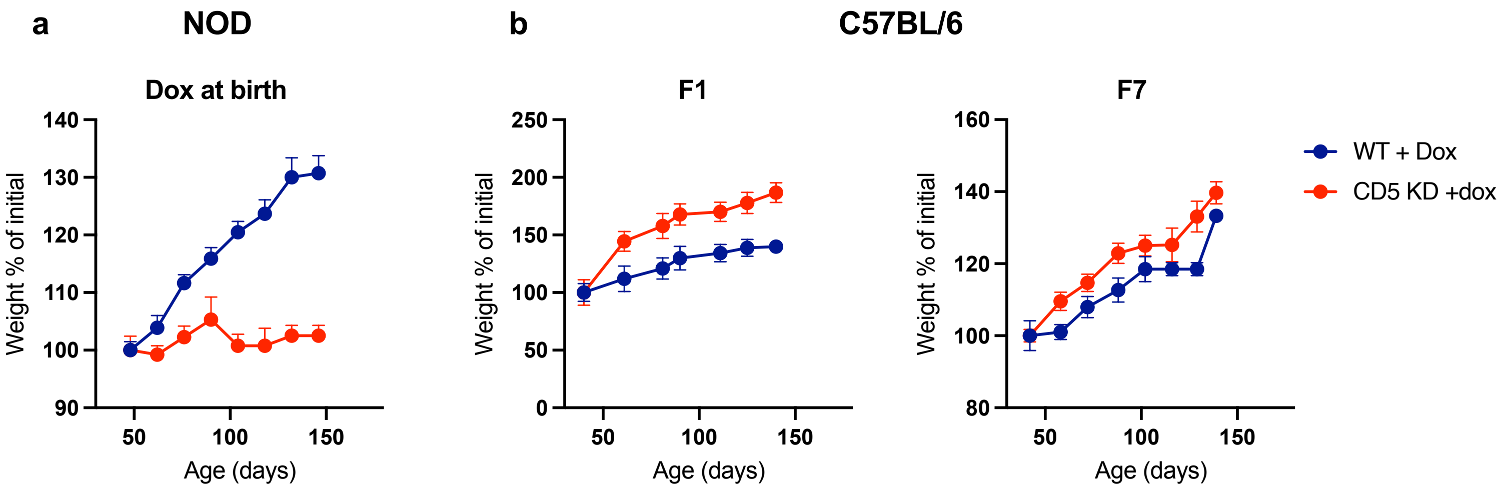

Supplementary Figure 2: CD5 KD does not significantly change the cellular composition of lymphoid tissues.

Frequency of $CD4^+$ T cells, $CD8^+$ T cells and B cells (a), $Foxp3^+$ $CD4^+$ T cells (b) and $CD44^{hi}CD62L^{lo}$ $CD4^+$ T cells (c) in the spleen, mesenteric lymph node (mLN) and Peyer's patches (PP) of dox-treated CD5 KD and WT NOD mice. were isolated and analyzed by flow cytometry. Representative FACS plots are shown on the left, cell frequency data (mean ± SEM) on the right. All mice were 7 weeks old, n=4-8 mice per group. (d) Representative images of spleens from dox-treated CD5 KD and NOD WT mice at 2 and 5 months of age as well as total splenic cellularity in 2m old mice. * $P < 0.05$ (two-tailed unpaired t-test).

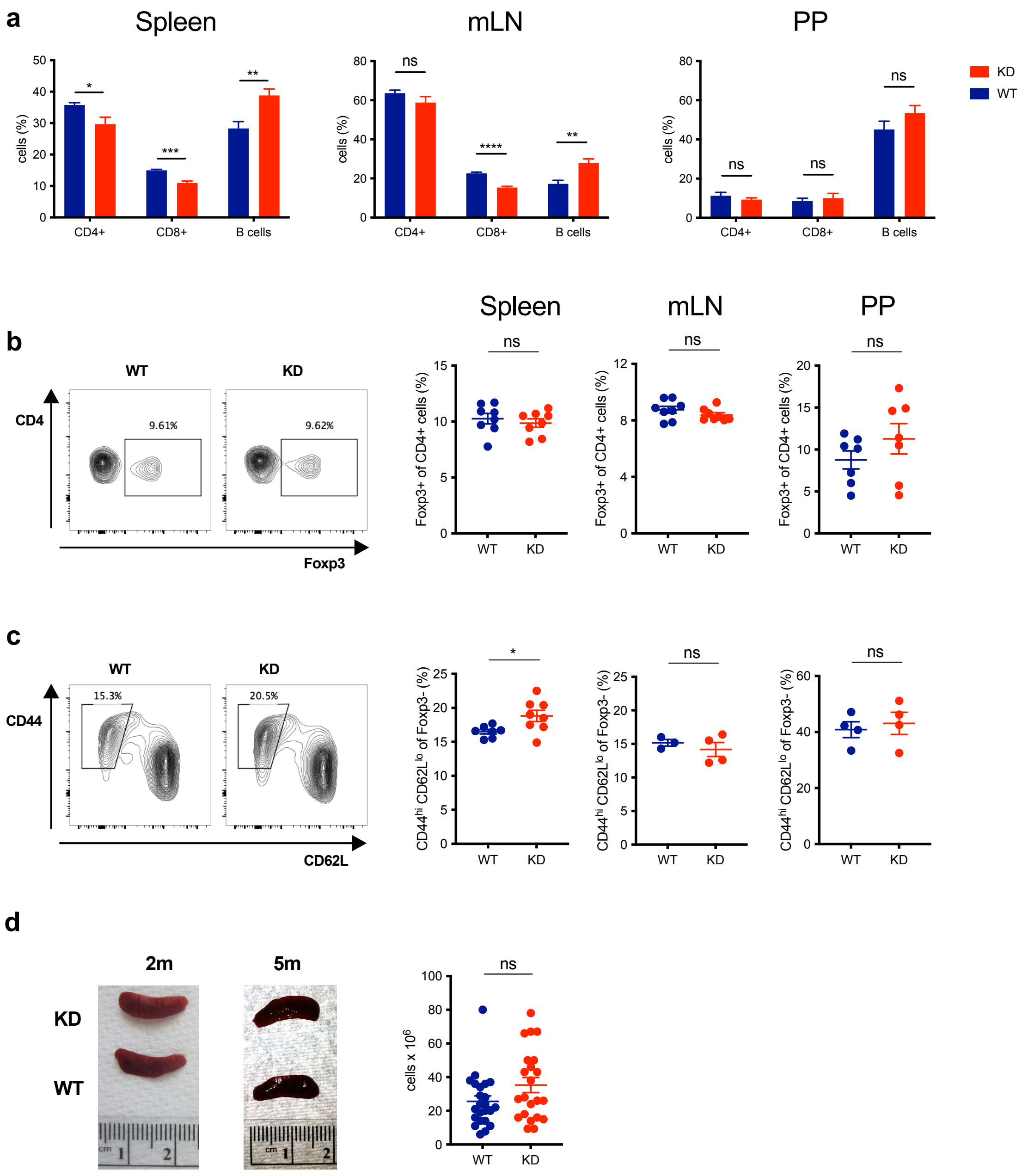

Supplementary Figure 3: CD5 KD does not significantly change IL-10 or TNF-α expression.

(a) IL-17A, IL-10 and TNF-α mRNA levels in $CD4^+$ T cells from WT^{dox} and CD5 KD^{dox} mice after *in vitro* Th17 (for IL-17A and IL-10) and Th1 (for TNF-α) differentiation.

(b) IL-17A and IL-10 mRNA levels in WT and CD5KD $CD4^+$ T cells isolated after adoptive transfer into gender-matched NOD.*scid* mice treated with doxycycline (n=5 mice per group).

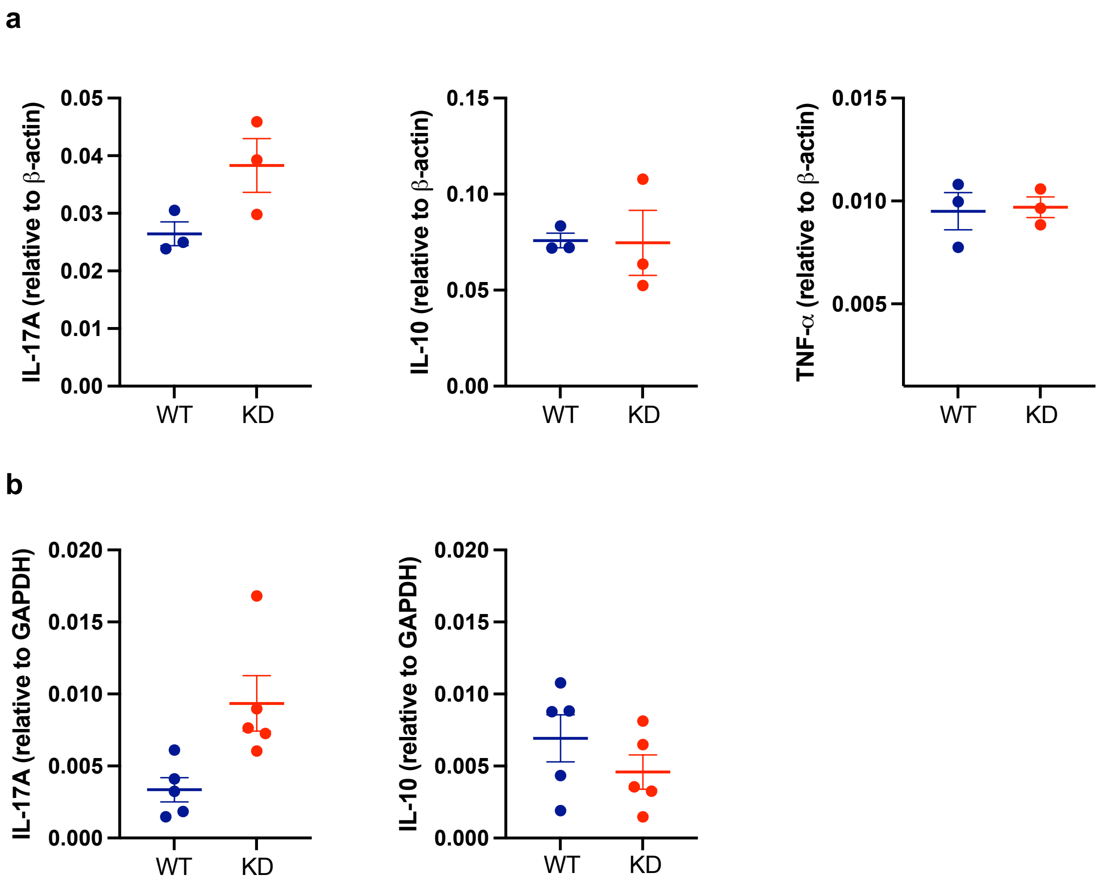

Supplement: Supplementary file 1 [file DataSheet_1.pdf]
